# A finite element method model to simulate laser interstitial thermo therapy in anatomical inhomogeneous regions

**DOI:** 10.1186/1475-925X-4-2

**Published:** 2005-01-04

**Authors:** Yassene Mohammed, Janko F Verhey

**Affiliations:** 1 Department of Medical Informatics, University of Goettingen, Robert-Koch-Str. 40, D-37075-Goettingen, Germany; 2 Department of Sciences and Technology, University of Applied Sciences and Arts, von-Ossietzky-Str. 99, D-37085-Goettingen, Germany

**Keywords:** LITT, laser induced thermotherapy, simulation, temperature calculation, perfusion, heat distribution, light diffusion, damage function, vessel, tissue, head-neck-region, minimal invasive therapy, therapy planning, tumour treatment

## Abstract

**Background:**

Laser Interstitial ThermoTherapy (LITT) is a well established surgical method. The use of LITT is so far limited to homogeneous tissues, e.g. the liver. One of the reasons is the limited capability of existing treatment planning models to calculate accurately the damage zone. The treatment planning in inhomogeneous tissues, especially of regions near main vessels, poses still a challenge. In order to extend the application of LITT to a wider range of anatomical regions new simulation methods are needed. The model described with this article enables efficient simulation for predicting damaged tissue as a basis for a future laser-surgical planning system. Previously we described the dependency of the model on geometry. With the presented paper including two video files we focus on the methodological, physical and mathematical background of the model.

**Methods:**

In contrast to previous simulation attempts, our model is based on finite element method (FEM). We propose the use of LITT, in sensitive areas such as the neck region to treat tumours in lymph node with dimensions of 0.5 cm – 2 cm in diameter near the carotid artery. Our model is based on calculations describing the light distribution using the diffusion approximation of the transport theory; the temperature rise using the bioheat equation, including the effect of microperfusion in tissue to determine the extent of thermal damage; and the dependency of thermal and optical properties on the temperature and the injury. Injury is estimated using a damage integral. To check our model we performed a first in vitro experiment on porcine muscle tissue.

**Results:**

We performed the derivation of the geometry from 3D ultrasound data and show for this proposed geometry the energy distribution, the heat elevation, and the damage zone. Further on, we perform a comparison with the in-vitro experiment. The calculation shows an error of 5% in the x-axis parallel to the blood vessel.

**Conclusions:**

The FEM technique proposed can overcome limitations of other methods and enables an efficient simulation for predicting the damage zone induced using LITT. Our calculations show clearly that major vessels would not be damaged. The area/volume of the damaged zone calculated from both simulation and in-vitro experiment fits well and the deviation is small. One of the main reasons for the deviation is the lack of accurate values of the tissue optical properties. In further experiments this needs to be validated.

## Introduction

Laser radiation is now used routinely in surgery to incise, coagulate, or vaporize tissues. The laser light power is converted into heat in the target volume with ensuing coagulative necrosis, secondary degeneration and atrophy, and tumour shrinkage with minimal damage to surrounding structures [1]. The use of lasers in surgery introduces some desirable features over normal surgical methods such as increased precision, improved hemostasis, and less tissue manipulation. Laser light power is thereby delivered to the targeted area by an optical fibre. The use of an optical fibre as applicator for interstitial light delivery was demonstrated, among others, by Bown [2] in 1983 as a method of heating and destroying deep-seated tumours [3]. The biological effects of laser energy depend on the laser wavelength, laser power, the duration of irradiance, blood perfusion, and both the optical and thermal properties of the tissue involved. Laser-tissue interaction mechanisms may be thermal, photochemical, or mechanical in nature [4]. Photochemical like PhotoDynamic Therapy (PDT). Mechanical like the effects induced using pulsed lasers (photoacoustic, photodisruptive). The surgical procedures that involve coagulation or ablation of tissue are thermal.

Clinical studies have yet to demonstrate that LITT is practical for the palliation of hepatic and nasopharyngeal tumours (e.g. [5-9]). The criteria for the clinical success of the thermotherapy for tumours in homogeneous tissues, for example, in the liver or brain, was described, among others, by Vogl [3], who placed applicator/diffuser at the centre of the tumour, using MRI online monitoring of the thermal changes to control the treatment [6]. In contrast to homogeneous and simple tissues, however, normal anatomical structures are more complicated. Especially for small tumours near main vessels a positioning of the laser applicator at the centre of the lesion is difficult and maybe not the optimum choice.

Modelling the laser-tissue interaction is beneficial for the analysis and optimisation of the parameters governing planned laser surgical procedures. Nevertheless, we still lack an adequate model that grants accuracy. Most of the models suggested depend greatly on simplifications of the real problem, either in the geometry they offer or in the system of equations they use. Some models, which use the bioheat equation, neglect the role of the changes in the tissue properties during temperature elevation process [10], which deem such a model unrealistic, especially considering high temperatures. Few modelling methods have simulated the behaviour of LITT in human tissue. Best known is the Monte-Carlo method described, among others, by Roggan et al. [11]. This method can simulate the use of multiple applicators but is limited to symmetric geometries and has not been correlated to real anatomic datasets. Similar to this is the finite difference method described in [12], where the authors did not include the coagulation process with its irreversible changes in optothermal tissue properties. In order to overcome these limitations, we included the damage function as well as perfusion terms in the modelling process, taking dependencies of these parameters into account.

This paper describes in detail the bases for a modelling method to simulate the effect of LITT for the treatment in various indications near large vessels, such as the carotid artery in the neck region. We thereby propose the use of LITT, frequently applied in the treatment of liver tumours [6], in more sensitive areas such as the neck region to treat tumours in lymph node metastases or epithelial carcinomas with dimensions of 0.5 cm – 2 cm in diameter.

The actual response of tissue to laser irradiation is a time-dependent phenomenon. Initially, there are thermal and possibly photochemical changes of the tissue at the molecular level. Next are changes in tissue perfusion caused by thermally induced vascular relaxation and/or vessel damage. Heat deposited at the application site is transferred to adjacent structures. This may be desirable for coagulation purposes – or it may cause unexpected thermal damage to otherwise viable tissues adjacent to the irradiation site. The rate of heat transfer depends on the composition and organization of tissues involved. Blood perfusion during and after irradiation has significant effects on the size of the damage zone.

We discuss in this paper our mathematical approach, its considerations and restrictions. In the main part we present the mathematical and physical backgrounds used to achieve the model. Then we present and discuss the results of our simulation in comparison with the results of our in-vitro experiment.

## Materials and methods

Our model of LITT considers both optical and thermal effects. It is based on calculations describing the light distribution using the diffusion approximation of the transport theory; the temperature rise using the bioheat equation, including the effect of microperfusion in tissue to determine the extent of thermal damage, and the dependence of thermal and optical properties on the temperature and the injury. Injury is estimated using a damage integral, which depends on the temperature elevation history. The order and flow of the modelling steps are described in the following sections in detail.

### The geometry of the 3D model

**Figure 1:**
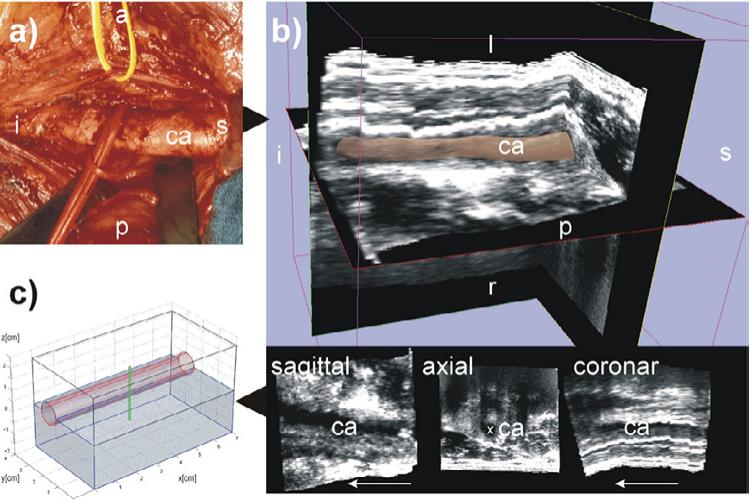
**The geometry used. **The left carotid artery is shown in this figure labelled with *ca*. The following letters indicate the orientation: s for superior, i for inferior, p for posterior, a for anterior, l for left, r for right. (a) is a photo of the human anatomy in the neck area. The carotid artery is shown here after moving the vein to the cranial direction. (b) shows the corresponding freehand 3D ultrasound dataset of the human neck region acquired axially. The 3D image in the top of (b) shows the 3D ultrasound volume together with the carotid artery segmented with 3D Slicer software [17]. The 3D model is displayed with 40% transparency. (c) displays the model used in the simulation approximated according to the geometry of the human neck shown in (a) and (b). The segmentation of the 3D ultrasound dataset in (b), is available as a video stream, too, showing the geometry of the carotid artery (see Additional file 1).

The head and neck area consists of complex anatomical structures in close proximity. In sonographic 3D volume datasets of the neck area the sternocleidomastoideus muscle and the neck vessels (common carotid artery, internal carotid artery, external carotid artery and the internal jugular vein) serve as leading structures [13]. Because of the almost superficial anatomic position of the vessels and their straight course (Fig. 1a) especially the common carotid artery is easily shown sonographically. Differentiation of inflammed lymph nodes and metastases located parallel to the large neck vessels [14] can be achieved 90% of the time with the help of signal-enhanced colour duplex sonography [15,16], making ultrasound preferable to other imaging techniques.

One can easily segment the carotid artery from 3D sonographical, MRI or CT volume datasets. For the segmentation of our 3D ultrasound dataset (Fig 1b) we used the software package 3D Slicer [17]. The real geometry is complex and needs to be simplified to reflect limited computational capacity. We thus obtained the 3D base model consisting of a cube of 4*4*7 cm^3 ^shown in Fig. 1c. The cube contains the blood vessel approximated either by the cylindrical shape or much better by a shape of cone. The applicator is shown as a thin tube of 2.5 cm perpendicular to the vessel (Fig. 1c).

Commercially available laser applicator fibres for thermotherapy frequently have a water jacket to cool the surface. The applicator is assumed to be a cylinder, and the cooling effect is implemented as a boundary condition at the diffuser surface.

**Figure 2:**
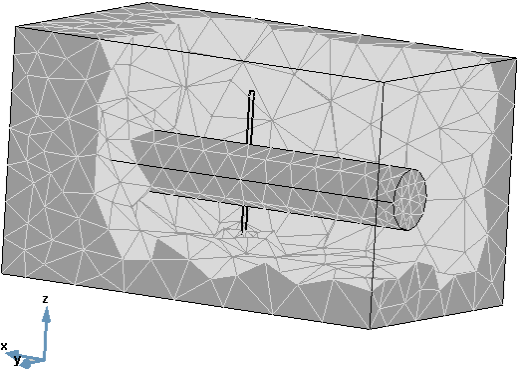
**The finite element method (FEM) mesh. **The values of each variable and of each property value are evaluated at each point of the mesh. In other words, the "simulation loop" in Fig. 3 is performed at each of point of the mesh. The bi-points variables' values are evaluated using an interpolation process.

The tissue surrounding the vessel is treated as a homogeneous muscle tissue. According to the geometry described using a mesh is generated to perform a finite element method calculation (Fig. 2). The model was implemented with FEMLAB 2.3 as an add-on to Matlab 6.5 for finite element modelling [18].

### The light distribution equation

In most tissues, both absorption and scattering are present simultaneously. A mathematical description of the absorption and scattering characteristics of light can be performed analytically or by using the transport theory. Transport theory has been extensively used when dealing with laser-tissue interactions. Furthermore, experimental results have confirmed its validity in most cases [19].

The photon propagation described using the transport theory has been dealt with already in [4,19-23]. Exact analytical solutions to the radiative transport equation have been found for only few special simple cases. However, when scattering processes dominate absorption in the medium, a high penetration depth of the light is the consequence. This is the case in LITT treatment in human tissue using an Nd:YAG 1064 nm laser or even a diode 830 nm laser: The penetration depth of both types ranges from between 1300 μm – and 1400 μm, whereas the penetration depth of other laser types like argon 514 nm laser or CO_2 _10600 nm laser is less than 350 μm. This leads us to the possibility of applying light diffusion approximation to the transport theory [4].

Because of the high penetration depth of the Nd:YAG laser in turbid media, diffusion theory provides a relatively accurate description of light propagation. In three dimensions the diffusion equation needs to be solved numerically, because an analytical solution is not possible [4]. FEM is the most practical method; moreover a number of different efficient solutions using FEM are now available.

**Figure 3:**
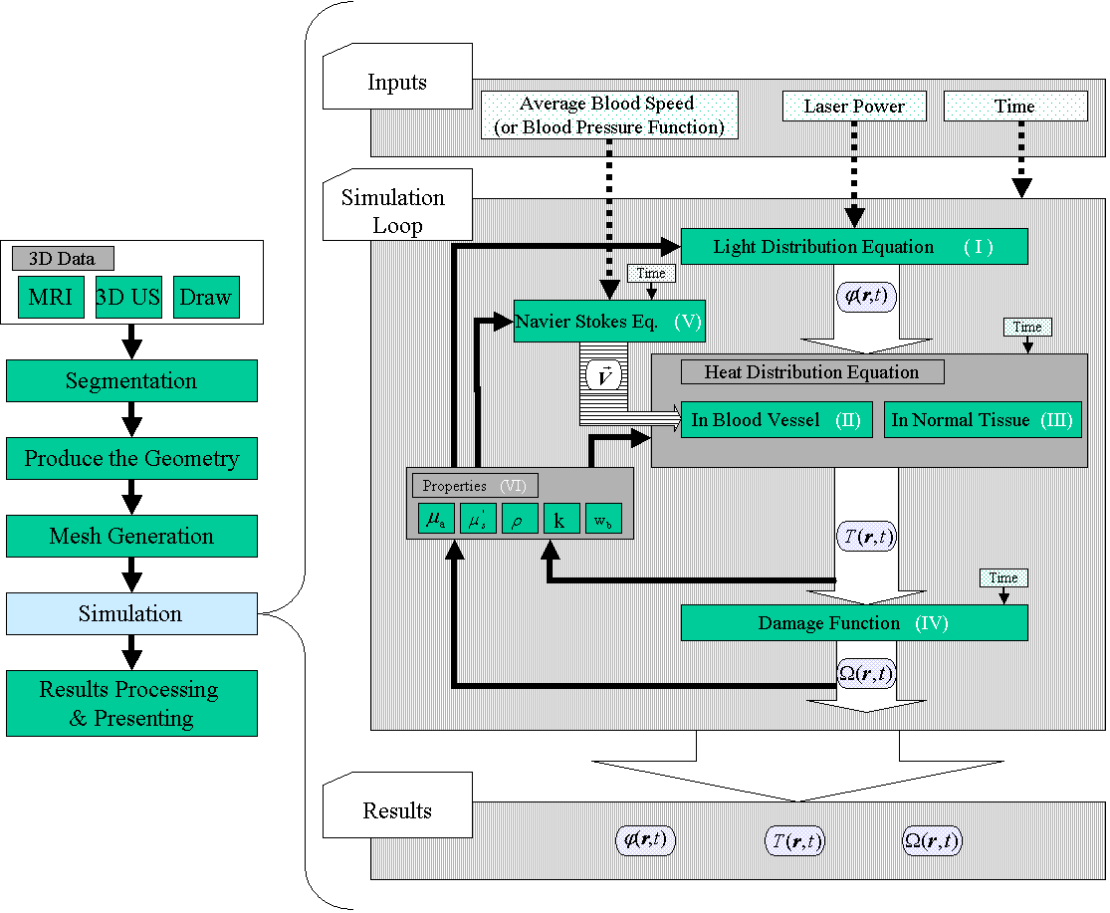
**The simulation loop. **The figure shows the simulation flow chart as a step in the imagined surgical planning system (left side). The future goal of the surgical planning system is to verify the three parameters governing a laser treatment: the applicator position, the laser power, and the exposure duration. The temperature starting point of the volume is set to normal body temperature. Three input parameters are taken: average blood velocity, laser power, and application time (right side: input). The main part of the simulation is the loop, which calculates the variables in the forward steps, then updates the values of the different properties (parameters) in the backward step according to the results of the forward step. The loop follows the section materials and methods and uses its nomenclature for variables and functions (right side: loop). The output of the solver consists of three parts: the light energy fluence rate *φ*(***r***.*t*), the temperature distribution *T*(***r***, *t*), and the damage Ω(***r***, *t*) (right side results). The results are explicity shown in figures Fig. 4 and Fig. 5. The roman numbers (I-VI) refer to the equations in the text.

An exact derivation of the light diffusion equation can be found in [4,20]. Here, we give the light diffusion approximation to the transport theory, which is implemented in our model (Fig. 3: Light Distribution Equation):



*φ *being the light fluence rate [W cm^-2^], *D *the diffusion coefficient [cm], and *Q *the source term [W cm^-3^]. *μ*_*a *_is the absorption coefficient and *μ*'_*s *_the reduced scattering coefficient in tissue. The roman number (I) indicates the position in Fig. 3. The relationship between the reduced scattering coefficient and the scattering coefficient, *μ*_*s *_is described by *μ*'_*s *_= *μ*_*s *_*(1-g)*, with g being the anisotropy factor incorporating the effects of directionally dependent scattering.

The absorption coefficient *μ*_*a *_for visible and for near infrared radiation ranges between 0.001 mm^-1 ^<* μ*_*a *_< 10 mm^-1 ^for biological tissues. While for the scattering coefficient *μ*_*s *_is in the order of 1 mm^-1 ^<*μ*_*s *_< 100 mm^-1 ^[20]. The optical properties *μ*_*a *_and *μ*'_*s *_depend on the tissue, and they change their values during a real treatment. In order to simulate this effect, the optical properties of the tissue are functions of the damage Ω [20] (see section on "damage function"). The damage function Ω describes the pathologic state of the tissue during treatment.

**Figure 4:**
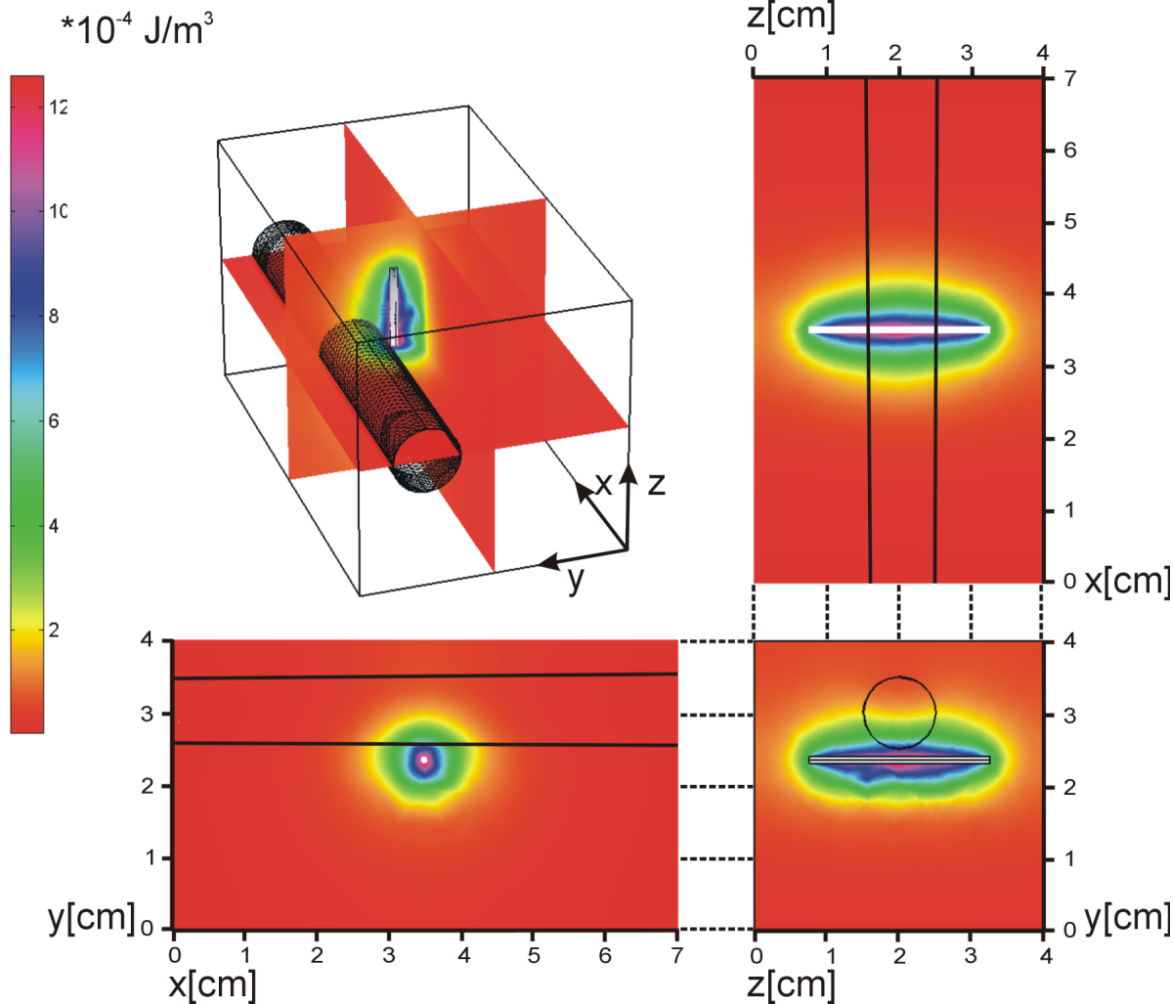
**Solution of the light distribution equation. **Left: the energy density colour index. The solution is elliptical as expected. The penetration depth in the vessel is less than in the tissue, because of different scattering and absorption coefficients.

In general, for simple geometries like a point-source, the solution of the light diffusion equation will be an exponentially decreasing function with effective attenuation coefficient given by:



As an example, the solution of eq. 1 for a light source similar to a medical applicator in a homogeneous medium takes the shape of an ellipsoid [24] as shown in Fig. 4.

### The heat distribution equation in tissue

The aim of irradiation with laser energy is to produce heat in the targeted tissue. Excess heat is either stored or extracted, leading to changes in the local temperature. The bioheat equation was repeatedly used to describe the heat changes in biological tissue [4,19,20]. The bioheat equation is the realizing of the principle of conservation of the energy applied to tissue volume, (Fig. 3: Heat Distribution Equation, in Normal Tissue),



where *T *is the temperature [°C], *ρ *the density of tissue [kg cm^-3^], *c *the specific heat of tissue [J kg^-1 ^°C^-1^], *k *the thermal conductivity of tissue [W cm^-1 ^C^-1^], ***r ***the position vector [cm], *t *the time [s], *T*_*art *_the temperature of arterial blood [°C], *S *the deposited light power [W cm^-3^], *w*_*b *_[ml/(g.min)] is the tissue average volumetric blood perfusion rate (but because the density of blood *ρ*_*b *_is to be considered as a constant value, it is possible to call *ρ*_*b*_·*w*_*b *_[kg s^-1 ^cm^-3^] the *average volumetric blood perfusion rate*, unfortunately usually denoted in the literature also with *w*_*b*_), and *c*_*b *_the specific heat of blood [J kg^-1 ^C^-1^]. The coefficients *ρ*, *k *and *w*_*b *_are functions of temperature *T*.

As a basis for the optical and thermal parameters for the simulations, we used values published by Mueller et al. [1]. Especially for normal body muscle tissue the physical properties are collected in Table 1.

**Table 1:** Listing of physical parameters. The table shows the physical parameters for native tissue and for coagulated-tissue, as well as for blood [25]. The small letters indicate the references where the material parameters are taken from: a) [20], b) [26], c) [27], d) [28], e) [29].

	Muscle		Blood
		
	Native	Coagulated	
Absorption coefficient, *μ*_*a *_(cm^-1^)	^a) ^0.23	^a) ^0.22	^b) ^0.44
Scattering coefficient, *μ*_*s*_' (cm^-1^)	^a) ^1.3	^a) ^13	^b) ^2.78
Density, *ρ *(kg cm^-3^)	^c), d) ^1.04·10^-3^		^d) ^1.06·10^-3^
Specific heat capacity, *c *(J kg^-1 ^°C^-1^)	^d) ^3.64·10^3^		^d) ^3.89·10^3^
Heat conductivity, *k *(W cm^-1 ^°C^-1^)	^c) ^5.18·10^-3^	^e) ^5.4·10^-3^	

### The heat distribution equation in large vessels

#### 1. The incompressible Navier-Stokes equation

In order to make the model adaptable to individual shapes of segmented vessels, we considered the geometry of a large vessel as a volume in which an incompressible fluid (blood) flows. The direction of the blood flow and the initial speed profile are implemented as boundary conditions. The incompressible Navier-Stokes equation for the blood (Newtonian fluid) reads:



Here, *η *is the dynamic viscosity [kg s m^-1^], *ρ *the density [kg m^-3^], *u *the velocity field, *p *the pressure [N/m^2^], and *F *a volume force field such as gravity.

Implementing the Navier-Stokes equation in the model allows us to present a time-periodic change in the blood flow rate, i.e., to simulate the beat cycle effect in the vessel. The main effect here on the result of the simulation lies in the accuracy of the estimated heat elevation in the tissue: A continuous blood flow has a different profile than the cycled flow (laminar and not laminar, or rather complex with four beat phases), which yields a different final cooling effect. For vessels away from the heart, the pumping cycle does not clearly appear; it tends to be a normal laminar flow. In this case (*u*(***r***, *t*) = *u*(***r***)) and eq. 5 is reduced to the following:



#### 2. The bioheat equation in large vessels

The heat convection between tissue and a large vessel occurs as a direct energy transfer rather than perfusion. The vessel is a heat sink in the treated volume. Therefore, the perfusion term in the bioheat equation has to be modified to consider heat conduction and blood flow. A new term, the so-called *enthalpy transport*, is added to retain the validity of the bioheat equation. The term serves for interpreting the internal energy flow out of the control tissue volume by means of the blood flow [30]. Considering the blood velocity field () in a large vessel the bioheat equation becomes:



### The damage function

The thermal damage in cells and tissue is described mathematically by a first-order thermal-chemical rate equation, in which temperature history determines damage. Damage is considered to be a unimolecular process, whereby native molecules transform into a denatured/coagulated state through an activated state leading to cell death [4,19]. Damage is quantified using a single parameter, Ω, which ranges on the entire positive real axis and is calculated from the Arrhenius law:



where *A *[s^-1^] is the frequency factor, *Ea *[J/mole] the activation energy, *R *[J mole^-1 ^K^-1^] the universal gas constant, and *T *[K] the temperature. C(***r***, 0) and C(***r***, *τ*) are the concentrations of the undamaged molecules at the beginning and at time *τ*, respectively. Damage Ω (eq. 8, Fig. 3, Damage Function) is dimensionless, exponentially dependent on temperature, and linearly dependant on time of exposure.

**Figure 5:**
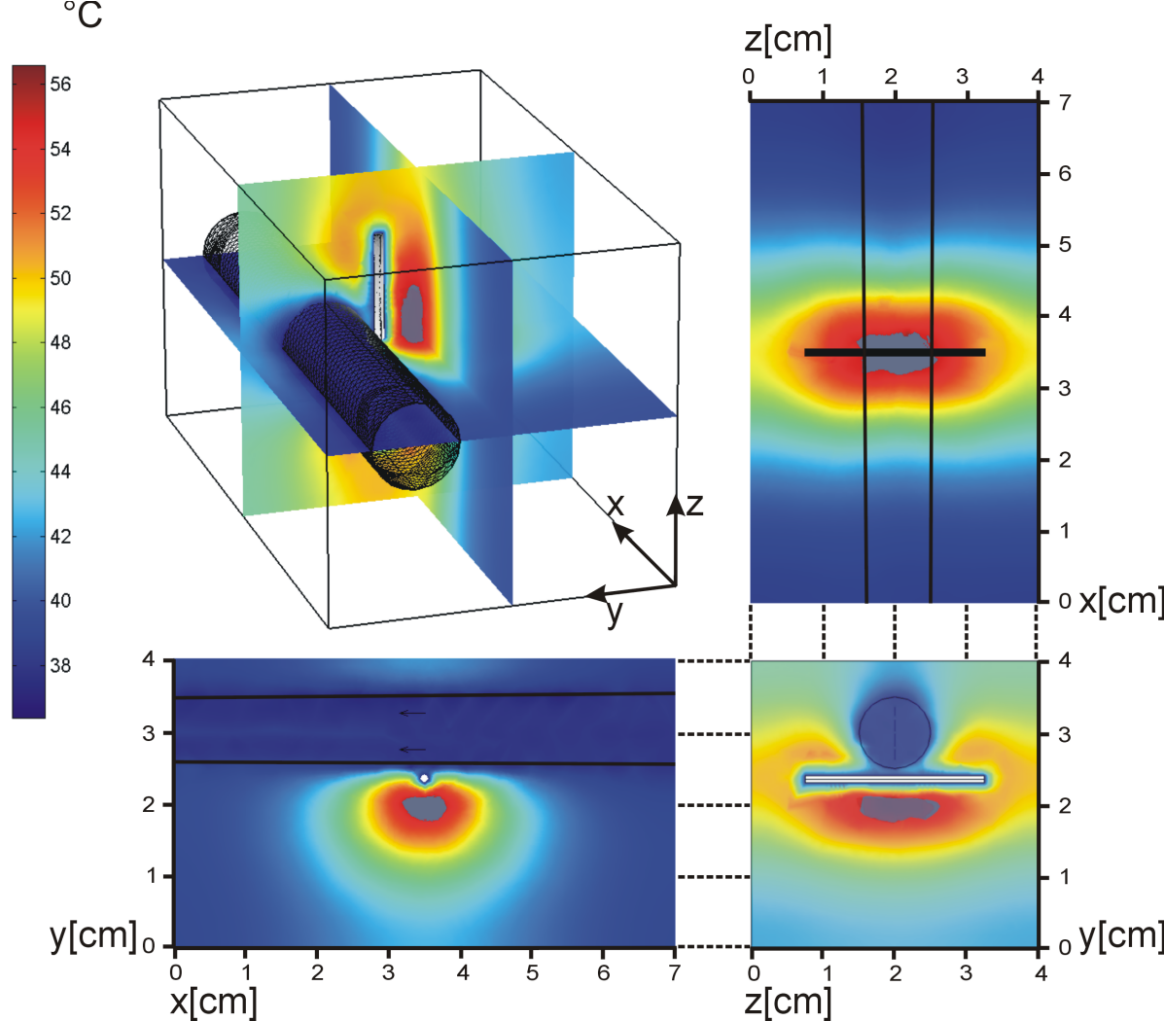
**The heat distribution and the damage in the volume. **Left: the heat colour index in °C. The damage appears when the value of the damage function Ω (eq. 8) reaches the threshold of 0.6. Here the image shows the results after 200 s. The damage zone is shown in grey. Fig. 5 is available also as a video stream demonstrates the temperature rise inside the tissue. The video stream shows where, how, and when this damage appears (see additional file 1).

The activation energy *E*_*a *_and the frequency factor *A *are derived from thermodynamic variables. They describe the denaturation process of proteins and other cellular constituents. *A *ranges from 10^40^s^-1 ^to 10^105^s^-1^, and *E*_*a *_from 10^5^J/mole to 10^6^J/mole [4]. The equation above indicates that the measure of damage describes the probability for tissue being destructed. It is the logarithm of the ratio of the initial concentration of undamaged tissue to the concentration after damage has accumulated, for the time interval *t *= 0 to *t *= *τ*. Therefore, Ω = 1 corresponds to the reduction in concentration of native molecules to a 37% level for a unimolecular system – an irreversible damage of 100% of the affected cells. However, in terms of the thermal damage to tissue, Ω (**r**, t) is a function of the observer's definition of damage. In [31] a limit of Ω >0.6 has been discussed as a margin of final tissue destruction (Fig. 5). A value of Ω = 0.6 corresponds to reduction in concentration of native molecules to 50% level.

### The dependences of the tissue properties on tissue temperature and damage

Heat capacity is assumed to be constant over a wide temperature range. The temperature dependence of thermal conductivity and density is taken into consideration by the following linear approximations [20] (Fig. 3: Properties):





On the other hand, the optical properties are influenced directly by the damage Ω. The scattering coefficient of coagulated tissue is much higher than that of native tissue. The optical properties change during the process of tissue coagulation, leading to a higher scattering coefficient and a nearly constant absorption coefficient. This becomes obvious by the change of the colour of the irradiated area (bleaching) and leads to reduction in penetration depth. The actual property set is calculated from the actual damage value as well as the optical properties in the native and coagulated tissue states [20] (Fig. 3, Properties):







Here, *μ*_*s*, *native *_and *μ*_*s*, *coagulated *_denote the scattering coefficients of native and coagulated tissue, respectively, *g *being the anisotropy factor. In literature *g *is mostly considered constant. Anyhow, some authors [20,31] reported the possibility of different values for *g *according to the damage state.

### Model implementation

The diagram in Fig. 3 illustrates the flow of the simulation. There are three main parts to the modelling of laser-tissue interaction (Fig. 3, right part):

• First, the irradiance distribution in the different tissues is determined by directly applying eq. 1. As shown in equations eq. 11, eq. 12, and eq. 13 the values of the properties depend on damage Ω (eq. 8). In the first loop step Ω is zero, and it starts to increase according to the rise in temperature, i.e., the different optical properties have as their starting point native tissue and as end point coagulated tissue. The actual value lies between both limits as determined according to Ω.

• In the second step, the temperature distribution in the tissue caused by laser energy deposition is estimated by solving the two bioheat equations for tissue and large vessel. The source term in both equations is defined by the absorbed energy at each mesh point (Fig. 2) from the distributed light energy calculated in the first step (light-energy to heat-source coupling) using:



• A by-step here is the estimation of the blood speed field () from the Navier-Stokes equation (either eq.5 or eq. 6). In our solved model, because we suggested treatment as taking place in the neck near the carotid vessel, we considered to use eq. 6 for obtaining the speed field, which is valid for laminar flow.

• In the third and main part, thermal damage is predicted from spatial and temporal temperature distribution (eq. 8).

• After estimating the heat distribution and the damage value, we perform a backward step to calculate the new values of the properties according to eq. 9 through eq. 12, which are updated in the equations set for the next loop iteration.

For our calculations we used FEMLAB's standard mesh generator with its default settings for modelling [18]. The mesh consists of 5354 nodes, more dense near the applicator and becomes coarser moving towards the walls. The time-dependent equation set described in the previous sections was solved using FEMLAB's time-dependent solver "femtime". The default settings for the solver were used: 0.01 for relative error tolerance and 0.001 for absolute error tolerance. Because of the non-linearity of this problem, the special time stepping algorithm "fldaspk" offered by FEMLAB was used in order to obtain a stable and convergent numerical solution [18]. A normal numerical solution to initial value problem of differential equation generates a sequence of values for the independent variable (time) *t*_*n *_and a corresponding sequence of values for the dependent variable (*φ*, *T*, *u*, Ω, and all other variables depend on them in this case) so that each *φ*_*n*_, *T*_*n*_,... approximates the solution at *t*_*n *_[32]. Modern numerical methods automatically determine the step sizes *h*_*n *_= *t*_*n*+1 _- *t*_*n *_so that the estimated error in the numerical solution is controlled by a specified tolerance [32]. The "fldaspk" solver uses the algorithm of the known DASPK solver written by Linda Petzold, which uses variable-order variable-stepsize backward differentiation formulas (for independent variable, time *t *in this case) [33]. There is no control on the time step itself, rather on the specified tolerance of each variable.

Light is considered to be emitted from an interstitial fibre with a fibre-diameter of 1 mm; it was modelled as an isotropically radiating cylindrical source (Fig. 4).

In the real treatment a cooling process using a special cooling catheter is performed to keep the temperature at the surface of the applicator low, preventing damage at its surface. A special boundary condition at the applicator surface should be applied in order to simulate this cooling effect. In the model this is realized by setting the outer surface temperature of the applicator to a constant value (normal body temperature T = 37°C).

At the modelled volume surfaces the insulation boundary conditions, optical and thermal, are used,



where ***n ***is the outward unit vector normal to the surface. This means the gradients of light fluence rate and of temperature vanish at the surface. Even though this condition is more suitable for light fluence rate, as small amount of radiations reach the surface, but in general the temperature and light fluence rate will not be constant. The need to set this condition in this way is because that the numerical solver demands defined fixed boundary conditions, which sometimes do not agree with the real situation.

According to the NCRP-data [34] the perfusion rate *w*_*b *_over the entie tissue is set to 1.4·10^-6^kg of blood s^-1 ^cm^-3 ^for *T *< 60°C and to 0 when *T *≥ 60°C, which is to be considered as a normal result of stopping the blood perfusion according to temperature elevation in the tissue [31]. In order to solve the Navier-Stokes equation, we set the dynamic viscosity, *η*, to 3.5·10^3^kg·s·m^-1^. To evaluate the damage, Ω, the activation energy *E*_a _is set to 670000 J/mol and the frequency factor A to 9.4·10^104^s^-1 ^[22,35]. Damage is considered to appear when Ω reaches the value of 0.6 [31].

The simulation takes around 2 hours on Sun Blade 2000 with Solaris 9 OS, 6 GB RAM and *Ultra*SPARC IIIi processor.

### Experimental validation

**Figure 6:**
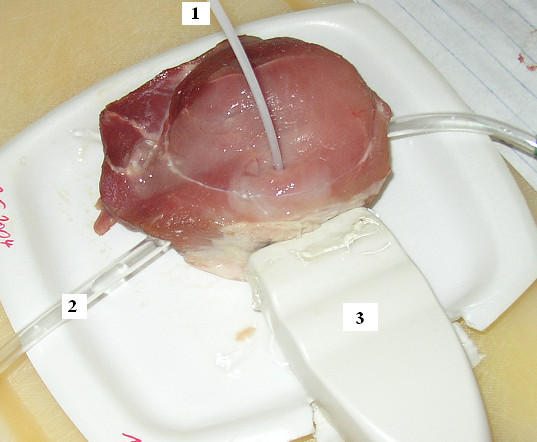
**The experiment setup. **1- laser applicator, 2- transparent tube (considered as a blood vessel), and 3- is ultrasound sonography prob. The tube was introduces by pulling it through a hole made using dipped knife. The online sonography was used to verify the position of the applicator.

We performed a single in-vitro experiment to check our model. The setup is shown in Fig. 6. The experimental work was performed using a fresh piece of porcine muscle tissue. In order to simulate the cooling effect of the blood flow in a vessel, a transparent tube (Polyethylene) and a porcine blood were used. An electronically controlled roller pump system (Storz Endomat LC203303) was used to pump the blood through the tube. We used the online ultrasound sonography to situate the applicator in its right position in front of the tube and to get the 3D ultrasound data analogy to the base geometry shown in Fig. 1. The sample has dimensions of 12*6*5 cm^3^. The tube inner diameter is 5 mm and the outer diameter is 7 mm. The blood and the laser cooling liquid had the room temperature of 21.4°C while the sample itself 17.6°C. A laser power of 30 W and blood average flow speed of 40 ml·s^-1 ^were used. We measured the exact distance between the laser applicator and the tube edge, 3 mm, at the end of the experiment after performing the cut in the sample. We fixed our application time to 300 s.

In the simulation model we omit the perfusion term, as there is no perfusion to be considered in-vitro. We simulated the tube (blood vessel) with diameter of 6 mm. All other experimental conditions are implemented in the model as they are in the experiment.

Because of the lack of data, which describe the properties of the porcine tissues, in all literature available to us, we used the same data presented in Table 1 to complete this simulation.

The highest temperature and widest damage are reached in front of the centre of the applicator. Taking into account the perpendicular surface to the applicator at centre, which is the most critical in the volume as all effects participate together: applicator, vessel, and blood flow, we complete the comparisons of the results between the experiment and the simulation using this plane. Hence, we made a cut in the probe at the level equivalent to this plane.

Coagulation of tissue is immediately apparent and always indicates lethal thermal effect [4]. Anyhow, and in order to comment on, a damage boundaries have to be identified. For most tissues, coagulation can be seen with naked eye as whitening of the tissue associated with turgor and opacity [4]. The damage boundary in the probe cut is determined by applying a grey threshold of 50% on the picture of the cut after changing it to grey scaled image. Away from the applicator position in a certainly undamaged area, applying this threshold led to 92.68% of the pixels to be black and 7.32% white, while in the damaged tissue 6.64% of the pixels were black and 93.3% white. The Matlab 6.5 R13, its Image Processing 4.0 Toolbox, and Paint Shop pro 8 were used to perform these steps.

A comparison in the z-direction needs an up-down cut (y-z plane) through the applicator position perpendicular to the tube/vessel, which was not possible after the cut in x-y direction.

## Results

In [25] we presented results from different geometries. Here, we focus on the results from the physical and mathematical points of view. We present also a comparison between the results of our model and the results of the in-vitro experiment.

### Theoretical results

Fig. 4 shows that the light energy is distributed, as expected, in a shape of ellipsoid, because of the absorption in the tissue. It also shows that the light penetration depth in blood is less than in the normal tissue, because of the different values of the absorption and scattering coefficients.

Fig. 5 shows the heat elevation. The cooling effect of the blood vessel may be clearly identified here. Fig. 5 is available as a video stream, too. This video stream demonstrates the temperature rise inside the tissue as a result of the irradiation. The video stream shows where, how, and when this damage appears (see Additional file 2).

The dimensions of the damage zone, which may be considered the target goal of the simulation, can be calculated directly by producing grided axes in all the 3D and 2D results as well as with routines written especially for this aim.

### Comparison with experimental results

**Figure 7:**
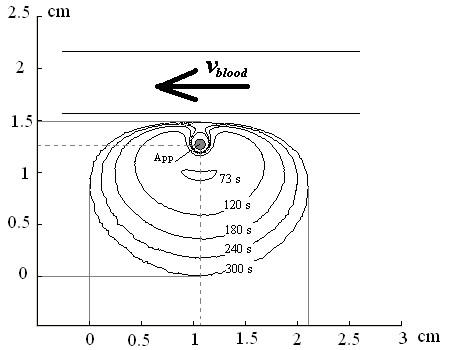
**The interpreted development of the damage zone as a result of the simulation model using the experimental conditions. **The applicator is recognized as a grey point in the middle and is indicated with (App). The damage zone starts with an application time of 72 s. The damage zone is presented here at 73 s, 120 s, 180 s, 240 s, and 300 s of application time as indicated in the figure.

Fig. 7 shows the interpreted development of the damage zone. The calculation shows that the damage starts with an application time of 72 s using laser power of 30 W. It starts at a distance of 2.5 mm from the centre of the applicator in the opposite direction of the vessel. After that, the damage zone will spread to all directions as it is shown in Fig. 7.

**Figure 8:**
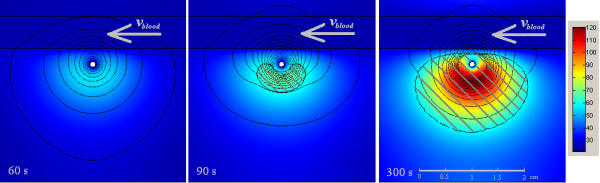
**Dispersion of heat and light distribution considering the experiment conditions. **The figure shows the results at 60 s, 90 s, and 300 s of application time. The isolines show the light distribution, which represent the deposition of the energy irradiated from the laser applicator. The major change occurred to the energy penetration depth happens between 60 s and 90 s of application time. The dashed area presents the damage zone at these times.

Fig. 8 shows the solution of the heat and light distribution equations for this model. In the first 60 s there is no noticeable change in the light distribution. In the next 30 s the damage zone appears, which leads to changes in the scattering and absorption coefficients (eq. 11 and eq. 12).

**Figure 9:**
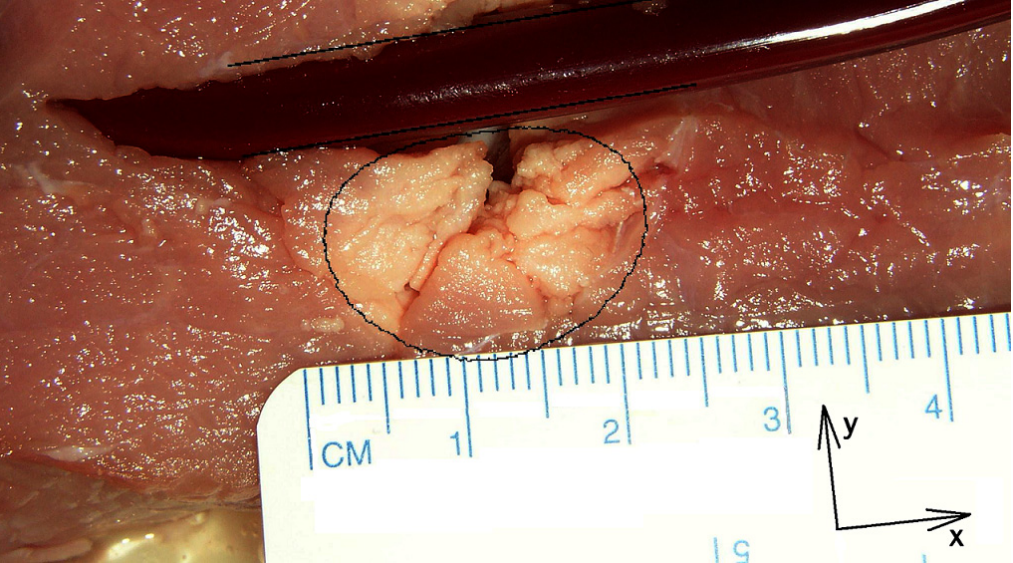
**The results from simulation and experiment. **For the experiment the following settings were used: time of application: 300 s, laser power: 30 W, blood flow rate: 40 ml/s, and applicator-vessel edge distance: 3 mm. Using these conditions a simulation is performed. In the figure the damage zones of both model and experiment are overlaid to show the deviation. The black oval shows the damage zone obtained from the simulation using the above conditions. The two parallel black lines indicate the vessel position and diameter in the simulation. The lesion is about 2*1.2 cm^2^, while the calculated damage zone is 2.1*1.45 cm^2^. The calculation error ranges from 5% in the x-axis parallel to the blood vessel to 20% in the y-direction perpendicular to the vessel. Both cutting the tissue with scalpel and the opening induced a tissue movement. This movement is a reason for the error in y-direction beside the error obtained from the optical properties, which affect all directions. The discoloration in the top of the image at the opposite side of the artificial vessel is due to the cut and the opening of the probe.

Fig. 9 shows the overlaid damage zones of both model and experiment. The kidney-shaped lesion is about 2*1.2 cm^2^, while our model shows a damaged zone of 2.1*1.45 cm^2^. We obtain calculation errors of 5% in the x-axis parallel to the blood vessel, and of 20% in the y-direction perpendicular to the vessel. This deviation happens mainly due to inaccurate optical properties values.

## Discussion

To date most simulation models of LITT have used the Monte-Carlo method (MC) to calculate the light distribution, then combine its results with Finite Difference method (FDM) to calculate the heat distribution. Because of its formulation, this combination fits very well for a radially symmetric problem. A weakness arises, however, when dealing with asymmetric volumes in real human anatomy. Arbitrarily curved surfaces separate the different tissues. Consequently, calculations from the FDM becomes so complex that errors start to appear in the results presented stemming from the dependency of FDM on dividing the volume into voxels. One way to overcome this is to increase the voxels' number. Indeed, this leads to less error at the tissue-separating surfaces, although it increases the resources and calculation steps, making the procedure inconvenient. In principle, combining MC and FEM (instead of FDM) is possible theoretically, and seems to be promising as it overcomes the latter problems, but to our knowledge has not implemented yet. From another perspective, MC solution converges to the exact solution of the transport equation only when the number of traced photons increases infinitely [4,21], which yield time consuming calculations.

Because we are dealing with an asymmetric geometry, we chose the FEM. It allows us to define and refine the mesh in the volume of interest in order to obtain more precise results. Furthermore, using a FEM mesh we are able to adapt individually the mesh for each patient's dataset. Never the less, it was not necessary to combine methods, FEM is used for all equations.

The model we propose depends on the following considerations:

• The coupling of a set of time-dependent equations, which simulate the whole process of the LITT treatment (Fig. 3). The set of equations includes the light diffusion equation, the bioheat equation, the Navier-Stocks equation, the damage function, and the dependencies of the properties on temperature and ensued damage.

• We consider the functional dependence of the various tissue properties at the various spatial and temporal points, according either to the tissue type, or temperature, or the damage value – or even a combination thereof.

• We take into account the irreversible changes in the tissue stemming from the treatment as they directly affect the solution of the set of equations.

Our model remains a mathematical model, meaning errors could appear from the considerations and simplifications made to realize it. Generally, such errors appear because of the following reasons:

• The inaccuracy of the optical, thermal, and damage properties are main point in the model's set of equations. In fact, these properties play a key role in the accuracy of the model's results. Many methods have been presented to calculate these properties [4,19,21], but still we see differences in the values presented by the different groups, which reflect the difficulty of measuring these properties. The problem is increased by the dependencies of the properties on the different variables (temperature, damage) over time. This makes the deviation neither linear nor regular.

• An error appears because of machine performance limitations: The available memory limits the number of mesh nodes and the degrees of freedom (DOF) used to build the model. This causes a deviation from an otherwise accurate result [18]. On the other hand, it is useless to increase the nodes number or the DOF arbitrarily: This would result in more time consuming calculations, since one would always have to run an interpolation and smoothing process as next to last step. In practice, a suitable number of nodes/DOF should be chosen, so that the interpolation routines estimate smoothly the value of all variables between nodes. Our model is based on what was mentioned above, with FEMLAB's standard refining processes at the critical areas (around the diffuser and vessel). It is important to refine the mesh around such surfaces, something that can be done much more conveniently using FEM rather than FDM. The convenience of the FEM-based modelling may be found in this very point of its ability to have different degrees of refining in the mesh according to how critical the region is.

• Absolute tolerance: All numerical methods have an allowed error (absolute tolerance) that reflects the criterion of the convergence. Normally, different solvers use different tolerances. In our model we used the FEMLAB's default tolerance value of 0.01 which leads to a final error of 1%, considered as a reasonable value for modelling.

One way to follow these errors and deviations from a real treatment result is to estimate them and to eliminate their effects from the final results of the model. This can be realized and implemented in the model by adding an error-correcting factor from the first degree (or even higher) in the set of equations correcting the result of each equation at each time step. These corrective factors should be measured practically by comparing the results of the model and the results from real experiments on test tissues or probes.

Our experiment shows a deviation of 5% in x-direction and 20% in y-direction. As the main reason for this deviation we propose inaccurate values for the optical tissue properties. Fig. 7 and Fig. 8 clearly show the kidney-shaped damage zone caused by the cooling effect of the blood vessel (or the tube in the experiment), which keeps its neighbouring in the native state for longer time. In literature [20,31], the value of the absorption coefficient *μ*_*a *_for both native and coagulated different biological tissues are close. Baring that in mind, and knowing that the value of the scattering coefficient *μ*_*s *_becomes normally, for biological tissue, 10 times greater than its starting value, i.e. native state, we can judge that as soon as the damage zone appears and the moving from native to coagulated state according to eq. 11, eq. 12, and eq. 13 the deviation in the calculations will increase as well.

Thus, accurate values of the different tissue properties, and especially the optical properties are key points in obtaining realistic results from the simulation. One promising technique for determination of optical properties was presented by Dam et al. [36]. There method provides an online values of the optical properties at 660 nm, 785 nm, 805 nm, 974 nm using a cylindrical probe head situated on the skin. It still require further researches. Anyhow, thinking of developing such a method to be able to gather information about optical properties at 1064 nm interstitially using a catheter during the irradiation can be of great value for modelling. Our model gives the possibility to implement such a gathered information directly. Thinking of using it online to predict the damage and controlling the irradiation power needs for sure more researches.

Finally, beside the error obtained from the optical properties, which affect all directions, both cutting the tissue with scalpel and the opening induced a tissue movement. This movement is a reason for deviation, especially in y-direction, as we perform the cutting in this direction.

## Conclusions

For several years now LITT has been a well-known and approved therapy system for tumour ablation in the liver and some other anatomical regions. Minimally invasive LITT procedures use a Nd:YAG 1064 nm laser. Therapy planning, however, remains unsolved and is still a challenging issue. Today's simulations are based on symmetric geometries. Without exact therapy planning systems, the usage of LITT is limited to homogeneous tissues or the respective surgeon's experience.

The finite element technique proposed in this paper can overcome both limitations. We propose a model to validate in the future the LITT method in other anatomic regions.

The model enables the efficient simulation for predicting the damaged zone induced with the diffuser of the LITT. The simulation is performed for tissue ablation near vessels, though obviously FEM is not limited to this. Exemplarily, we implemented the model for tissue ablation near the carotid artery in the neck region using an approximation for the artery shape. We describe the bases necessary to calculate the effects of the temperature rise caused by the absorption of light energy in the tissue, using the bioheat equation and including the cooling effects of vessel blood flow and micro-perfusion in tissue in order to determine the extent of thermal damage. The shape of the carotid artery is derived from a real segmented geometry based on, but not limited to, 3D ultrasound.

Experimentally, we performed a laser irradiation in a porcine muscle tissue sample. The results of our model diverge between 5% to 20% from the lesion obtained in the experimental work. From the authors' point of view two major reasons can be identified. The lack of accurate data describing the thermal and optical properties leads definitely to deviations. Furthermore the cut of the probe with scalpel induces a certain tissue shift, especially in the y-direction.

Anyhow, more experiments with different conditions are necessary to be able to carry out a statistical study and find the exact origin of the deviation, and, if necessary, define an error correction factors and add them to equation set. But that does not set aside the desire of accurate values for the properties of the tissue.

From another hand, still our model practical, it presents a step in using segmented data as basis for much more detailed surgical therapy planning. Combining LITT and adequate planning system could increase both the anatomical application range and the quality of therapy procedures.

## Supplementary Material

Additional File 1 **Animated gif file, the Geometry**. The animated gif shows the 3D ultrasound volume together with the carotid artery segmented using 3D Slicer software [17]. The movie belongs to Fig. 1b. The gif file can be played using the internet browser. Click here for file

Additional File 2 **Animated gif file, The heat distribution and the damage zone in the volume**. The video stream demonstrates the temperature rise inside the tissue. The video stream shows where, how, and when this damage appears. The damage zone is shown in grey colour. The gif file can be played using the internet browser. Click here for file
