# Supplementary figures and images for: A finite element method model to simulate laser interstitial thermo therapy in anatomical inhomogeneous regions
Source: Biomed Eng Online. 2005 Jan 4;4:2. doi: 10.1186/1475-925X-4-2 (PMC546235; doi:10.1186/1475-925X-4-2)

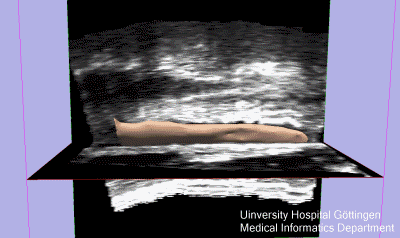

Supplement: Additional File 1 — Animated gif file, the Geometry. The animated gif shows the 3D ultrasound volume together with the carotid artery segmented using 3D Slicer software [17]. The movie belongs to Fig. 1b. The gif file can be played using the internet browser. [file 1475-925X-4-2-S1.gif]

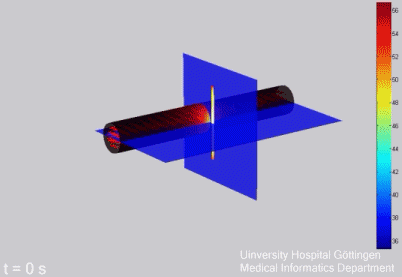

Supplement: Additional File 2 — Animated gif file, The heat distribution and the damage zone in the volume. The video stream demonstrates the temperature rise inside the tissue. The video stream shows where, how, and when this damage appears. The damage zone is shown in grey colour. The gif file can be played using the internet browser. [file 1475-925X-4-2-S2.gif]
